# Gene conversion: a non-Mendelian process integral to meiotic recombination

**DOI:** 10.1038/s41437-022-00523-3

**Published:** 2022-04-07

**Authors:** Alexander Lorenz, Samantha J. Mpaulo

**Affiliations:** Institute of Medical Sciences (IMS), University of Aberdeen, Aberdeen, UK

**Keywords:** Eukaryote, Genome

## Abstract

Meiosis is undoubtedly the mechanism that underpins Mendelian genetics. Meiosis is a specialised, reductional cell division which generates haploid gametes (reproductive cells) carrying a single chromosome complement from diploid progenitor cells harbouring two chromosome sets. Through this process, the hereditary material is shuffled and distributed into haploid gametes such that upon fertilisation, when two haploid gametes fuse, diploidy is restored in the zygote. During meiosis the transient physical connection of two homologous chromosomes (one originally inherited from each parent) each consisting of two sister chromatids and their subsequent segregation into four meiotic products (gametes), is what enables genetic marker assortment forming the core of Mendelian laws. The initiating events of meiotic recombination are DNA double-strand breaks (DSBs) which need to be repaired in a certain way to enable the homologous chromosomes to find each other. This is achieved by DSB ends searching for homologous repair templates and invading them. Ultimately, the repair of meiotic DSBs by homologous recombination physically connects homologous chromosomes through crossovers. These physical connections provided by crossovers enable faithful chromosome segregation. That being said, the DSB repair mechanism integral to meiotic recombination also produces genetic transmission distortions which manifest as postmeiotic segregation events and gene conversions. These processes are non-reciprocal genetic exchanges and thus non-Mendelian.

## Introduction

**Fig. 1:**
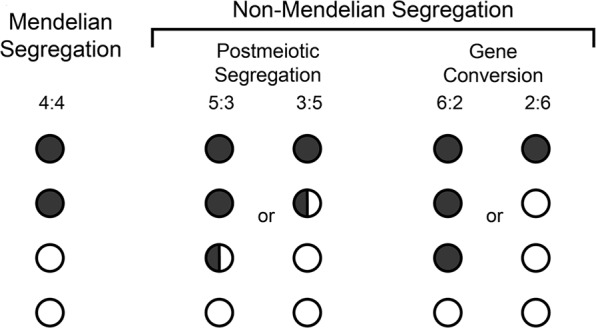
Schematic showing the Mendelian 4:4 segregation pattern, as well as the non-Mendelian 5:3/3:5 (PMS) and 6:2/2:6 (gene conversion) segregation patterns of a single heterozygous site. Recombination performed nearby such a heterozygous site will generate heteroduplex DNA (hDNA) containing mismatches which can lead to non-Mendelian segregation events.

**Fig. 2:**
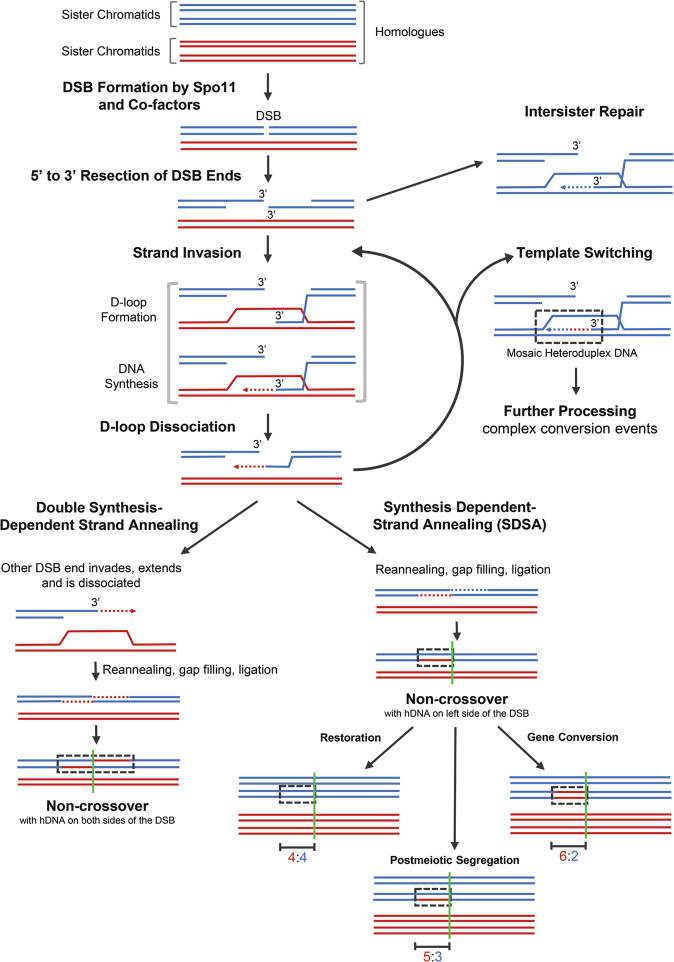
Models of intersister and non-crossover DSB repair pathways during meiosis. A homologous chromosome pair is represented by blue and red sister chromatids. For clarity only the chromatids involved in recombination are shown, except for the initial step of the pathways and the final step of canonical Synthesis-Dependent Strand Annealing (SDSA). Please, note that the small gap representing the DSB does not indicate loss of genetic material as with Spo11 double cutting (see Fig. 3). Early steps of recombination are shown: DSB formation by Spo11, DNA strand resection to expose 3’ single-stranded tails which then invade a homologous template to form Displacement loops (D-loops). D-loops can be dissociated (by the action of DNA helicases; reviewed in Lorenz 2017) before or after DNA synthesis has started; antirecombination driven by MutSα, MutSβ, and MutLα also plays a key role here (see main text). Canonical SDSA is thought to produce non-crossover gene conversion events. The position of the initiating DSB site is indicated by a green vertical line. Dependent on the actions of mismatch repair a given heterozygous site within hDNA can be left unrepaired (PMS, 5:3 segregation), converted (gene conversion, 6:2 segregation), or restored (Mendelian 4:4 segregation) (see main text and Fig. 1 for details). Multiple invasion/dissociation cycles can result in complex conversion events when template switches between sister chromatids and homologues occur. The simultaneous or consecutive invasion of both ends of the DSB into the same or different chromatids of the homologue can result in double SDSA, were conversion tracts left and right of the original DSB site can be detected.

Meiosis is a specialised type of cell division that results in the production of gametes (reproductive cells). During meiosis diploid progenitor cells undergo two sequential rounds of division without an intervening round of DNA replication. As a consequence, the hereditary material is halved to produce gametes, which are haploid meiotic products. Gametes in turn fuse during fertilisation forming a zygote which, like the parents, is diploid. During meiosis, chromosomes are also re-assorted and recombined, so that the gametes formed contain new chromosome configurations. The independent assortment of whole chromosomes already generates genetic diversity in the gametes. However, to shuffle the hereditary material within a chromosome, deliberate breakage of chromosomes (DNA double-strand breaks, DSBs) by the DNA topoisomerase-II-related transesterase Spo11 and subsequent repair into new combinations is required (Lam and Keeney 2015; Zickler and Kleckner 2015; Hunter 2015) (Figs. 1 and 2). To halve the hereditary material accurately, homologous chromosomes (homologues) must be segregated faithfully from each other in the first meiotic division. Damaging the hereditary material through the formation of DSBs is potentially hazardous. However, inducing multiple DSBs on each chromosome allows the homologues to find each other when DSB ends start searching for homologous repair templates (in invertebrates initial homologue recognition and pairing is protein-mediated and independent of DSBs) (Zickler and Kleckner 2015). In the end, the DSB repair by meiotic homologous recombination physically connects the homologues via crossovers. These physical connections enable faithful chromosome segregation. Since a single crossover connecting each homologue pair is sufficient for guiding correct chromosome segregation (Kan et al. 2011), the majority of DSBs are actually repaired as non-crossovers using the homologue as a template, or redirected for repair using the sister chromatid. Moreover, the process of meiotic homologous recombination is important for evolution, because it increases the genetic diversity in populations. This in turn promotes the fitness of natural populations, since it enables various features of parents to be distributed to their progeny in novel combinations. Indeed, meiotic chromosome segregation and meiotic recombination are at the core of what we call the Mendelian laws of heredity (Fig. 1). These two processes mechanistically enable the law of segregation and the law of independent assortment, and they also underpin Thomas Hunt Morgan’s chromosomal theory of inheritance (Bateson 1909; Morgan 1910). However, the repair of DSBs during meiosis also leads to outcomes which are non-Mendelian in nature: postmeiotic segregation and gene conversion (Fig. 1). (Please note, that gene conversion is not restricted to meiosis, it can also occur in vegetative cells during homology-directed DNA repair. This, however, will not generate genetic transmission distortions in reproductive cells). In this review, we discuss how these non-Mendelian events were discovered and what we understand about the molecular processes generating them.

According to the ‘Glossary of Genetics’ (Rieger et al. 1991), gene conversion is “the nonreciprocal recombinational transfer of genetic information between homologous DNA sequences (allelic or homologous nonallelic genes) without an accompanying information exchange.” Indeed, the term “Genkonversion” (German for gene conversion) was coined by Hans Winkler in 1930 to explain meiotic recombination outcomes in fungi (Brunswik 1926) and mosses (von Wettstein 1924), which were incongruous with crossing over events (Winkler 1930; Lindegren 1949, 1958). Presumably, because Winkler originally pitted his gene-conversion theory directly against Morgan’s crossing-over theory (Morgan 1910), and because the main discerning feature of a gene conversion was that it could not be explained as a crossover (Winkler 1930; Lindegren 1949, 1958; Rieger et al. 1991), some authors use the terms gene conversion and non-crossover synonymously; this is particularly an issue in the older literature. However, a few decades after the publication of Winkler’s gene-conversion theory (Winkler 1930), further work in the ascomycetes *Neurospora* and *Saccharomyces* established that gene conversions can also be associated with crossovers, and that these recombination outcomes are mechanistically linked rather than mutually exclusive (Case and Giles 1958; Whitehouse and Hastings 1965; Fogel and Hurst 1967); this is now widely accepted (Fogel et al. 1979; Szostak et al. 1983). Much of what we understand about the molecular mechanisms underpinning the formation of gene conversion, comes from research in the two model yeast species *Saccharomyces cerevisiae* and *Schizosaccharomyces pombe* (see below). Although both species are unicellular fungi (yeasts), they are actually not closely related to each other, as they had their last common ancestor roughly at the same time as nematodes and mammals had theirs (Heckman et al. 2001). This already strongly indicates that gene conversion is a highly conserved process. Indeed, gene conversion has also been described in several multicellular eukaryotic species, including *Drosophila melanogaster* (e.g. Miller et al. 2012; Comeron et al. 2012), *Arabidopsis thaliana* (e.g. Sun et al. 2012; Drouaud et al. 2013; Wijnker et al. 2013), mouse (e.g. Cole et al. 2010, 2014; Gergelits et al. 2021), and human (e.g. Reiter et al. 1998; Jeffreys and May 2004). *Caenorhabditis elegans* is the only notable model for meiosis research, in which gene conversion between homologous chromosomes during meiotic recombination has not been demonstrated. However, gene conversion between homologues is likely to occur, as it does happen in the *C. elegans* germline during transposon excision (Robert et al. 2008) and between sister chromatids (Almanzar et al. 2021; Toraason et al. 2021).

After their formation by Spo11, meiotic DSBs undergo 5’→3’ resection of the DSB ends on one DNA strand to expose the other DNA strand as a 3’ single-stranded tail, which then invades homologous DNA sequences to mend the broken chromosome (Fig. 2) (Cejka and Symington 2021). There can be DNA sequence differences between homologous chromosomes, especially in natural populations where homologues are rarely identical. These DNA sequence differences will create mismatches within the recombination intermediates produced by the strand invasion process, so called heteroduplex DNA (hDNA) (Surtees et al. 2004; Spies and Fishel 2015) (Figs. 1 and 2). The fate of hDNA determines genetic recombination outcome essentially in three ways (Surtees et al. 2004; Spies and Fishel 2015). Firstly, if hDNA is left untouched the result is postmeiotic segregation (PMS), this is seen as a 5:3 segregation of the involved DNA sequence differences in the progeny (Figs. 1 and 2). Secondly, if hDNA is mismatch-corrected towards the information on the invading strand (donor) the result is gene conversion (6:2 segregation) (Figs. 1 and 2). Thirdly, if the mismatch is corrected using the information on the invaded strand (acceptor) then a 4:4 segregation to the parental situation is restored (Figs. 1 and 2). PMS is rare in wild-type crosses of budding yeast and fission yeast, but happens at a much higher frequency in the absence of functional mismatch repair (Schär and Kohli 1993; Alani et al. 1994; Hunter and Borts 1997; Schär et al. 1997).

**Fig. 3:**
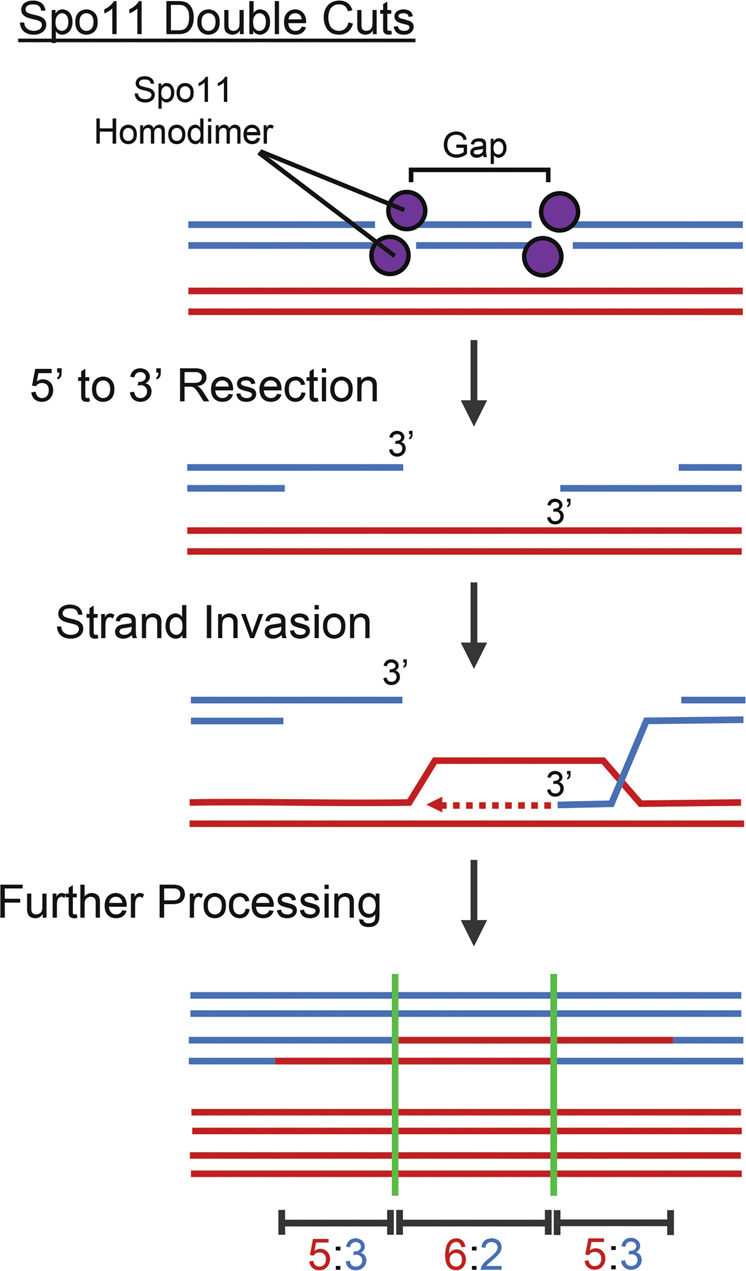
Model of direct formation of gene conversion (stretch of 6:2 segregation) and PMS (stretches of 5:3 segregation) at a gap created by two Spo11 DSBs in close proximity to each other. For the sake of simplicity only a single sister chromatid (one in blue, one in red) per homologous chromosome is shown, except for the last step to illustrate the segregation patterns of the recombination outcome. The positions of the initiating DSB sites are indicated by green vertical lines.

It has been suggested that full gene conversions can be generated independent of mismatch repair through the formation and repair of gaps at the DSB site (e.g. Szostak et al. 1983; Martini et al. 2011). Indeed, in mitotic cells the 3’ single-stranded DNA tail produced during DNA resection is unstable, and its shortening results in gapped DSBs (Zierhut and Diffley 2008). It is unclear whether this also occurs at Spo11-generated meiotic DSBs. However, it has recently been demonstrated that the formation of two meiotic DSBs by Spo11 on the same chromosome in close proximity (‘double cuts’), will generate gaps of ~30 – ~100 base pairs in size (Fig. 3). Such events are apparently quite common (1/5th of all DSB events), and their repair can directly result in 6:2 and 2:6 conversions (Fig. 3) (Johnson et al. 2021; Prieler et al. 2021).

Going forward, we will focus on gene conversion as the main non-Mendelian outcome of meiotic recombination in a wild-type setting.

## How do we detect and measure non-Mendelian segregation events?

Measuring gene conversion frequency at single nucleotide polymorphisms, small confined marker genes, or genetically engineered marker constructs, depends on several factors. Gene conversions tend to be infrequent events, which makes their detection difficult. This is especially the case, when one parent contains a wild-type marker gene and the other parent has a single DNA change. Here, gene conversion can only be detected in so-called tetrad dissection experiments. Tetrad dissection enables the analysis of all 4 products of a single meiosis revealing the aberrant 3:1 or 1:3 (6:2 or 2:6) segregation patterns of wild-type vs. mutant marker genes (Winkler 1930; Lindegren 1958). Dissecting hundreds, if not thousands, of tetrads to observe a few gene conversion events is laborious. If half-chromatids need to be studied to discern gene conversion from PMS events, this becomes even more involved. There are two biological features exploited in the last few decades which alleviated some of the challenges around measuring gene conversion frequency at specific markers. One is the discovery of so-called meiotic recombination hotspots (simply referred to as hotspots from here onwards), and the other is the use of 2-point or bifactorial crosses.

Hotspots are DNA sites or regions with a higher-than-average frequency of meiotic recombination (Petes 2001; Wahls and Davidson 2012). In the two model yeast species, there are natural hotspots (e.g. Lichten and Goldman 1995; Cromie et al. 2005; Steiner and Smith 2005a), hotspots generated by point mutations (e.g. Ponticelli et al. 1988; Schuchert and Kohli 1988; Kon et al. 1997), and biotechnologically engineered hotspots (e.g. White and Petes 1994; Baur et al. 2005). The frequency of recombination events, including gene conversions, at hotspots is largely defined by the amount of DSBs made by Spo11 within the hotspot region (Petes 2001; Lam and Keeney 2015). Interestingly, it has also been shown in *S. cerevisiae* that hypomorphic mutants of *SPO11* can affect gene conversion tract length differentially between non-crossover and crossover outcomes (Rockmill et al. 2013). This indicates that the extent of conversion at a specific locus can be misestimated, especially when it only contains a few scorable markers. Generally, the frequency of gene conversion will be higher at sites which receive large numbers of DSBs; this then requires fewer tetrad dissections to obtain meaningful data.

The second technical improvement is looking at 2-point or bifactorial crosses. Rather than studying gene conversion in crosses where one parent carries a mutant allele and the other a wild-type allele (1-point or monofactorial crosses), in bifactorial crosses parents with two different alleles (heteroalleles) at the same test locus are crossed with each other. Bifactorial crosses have a distinct advantage in that since both parents are mutant, only progeny having undergone gene conversion can be wild-type for the associated phenotype. Therefore, this circumvents the need to perform tetrad dissection to identify gene conversion events unambiguously (e.g. Gutz 1971; Schär et al. 1993; Zahn-Zabal and Kohli 1996). One pair of genes involved in adenine metabolism in both model yeasts, proved particularly useful for measuring gene conversion frequency in bifactorial crosses. These are *ADE1* (coding for the SAICAR synthetase) and *ADE2* (coding for the AIR carboxylase) in *S. cerevisiae*, and their orthologues in *Sz. pombe*, *ade7*^+^ and *ade6*^+^, respectively (Juang et al. 1993; Rébora et al. 2001). Mutations in these genes produce yeast colonies displaying a pink to red colour when grown under specific conditions. This makes it very easy to phenotype progeny which have undergone gene conversion restoring the wild-type creamy white colony colour, from bifactorial crosses where both parents carry mutant heteroalleles of these genes and thus form pink or red colonies (Lindegren 1949; Leupold 1958).

In *Sz. pombe*, identification of the *M26* mutation in *ade6* as a meiotic recombination and DSB hotspot (Schuchert and Kohli 1988; Steiner et al. 2002) enabled recombination frequency analysis at a hotspot in bifactorial crosses. Further improvements, including screening for hotter versions of the *ade6-M26* allele (Steiner and Smith 2005b), and generating a genetic interval with scorable marker genes around *ade6* to measure crossover frequency associated with gene conversion (Osman et al. 2003; Lorenz et al. 2010), make this a genetic tool used to this day.

Progress with high-density DNA microarray and whole-genome sequencing technologies also facilitated the mapping of recombination events in *S. cerevisiae* on a genome-wide scale. Here, 2 different strains of *S. cerevisiae* which harbour thousands of different natural genetic variants (single nucleotide polymorphisms, and small insertions & deletions) are crossed to each other, and the shuffled distribution of the genetic variants in the progeny of such hybrid meioses can be used to analyse gene conversion and PMS, as well as crossover and non-crossover frequencies (e.g. Chen et al. 2008; Mancera et al. 2008, 2011; Martini et al. 2011; Rockmill et al. 2013; Oke et al. 2014; Marsolier-Kergoat et al. 2018; Cooper et al. 2021; Johnson et al. 2021). Up to 1% of the genome can be subjected to gene conversion in each meiotic product (Mancera et al. 2008), indicating that the contribution of meiotic gene conversion to genetic diversity in progeny is substantial, but tends to be underestimated in comparison to crossovers (Cole et al. 2012).

The mismatch repair pathway governed by the MutSα (Msh2-Msh6), Mutsβ (Msh2-Msh3), and MutLα (Mlh1-Pms1) complexes is a major determinant of meiotic recombination outcome (Surtees et al. 2004; Spies and Fishel 2015). These particular MutS and MutL complexes influence meiotic recombination outcome on two levels (Surtees et al. 2004; Spies and Fishel 2015). Firstly, they repair mismatches in hDNA, thus driving restoration and conversion (Fig. 2). Secondly, MutS coordinates disassembly of recombination intermediates containing (too many) mismatches to ensure that repair is performed from a homologous template; this is called antirecombination. Mutants inactivating these functions of MutSα and MutLα thus serve as tools to enable the detection of hDNA and measure the length of DNA tracts in non-Mendelian segregation events. Notably, *S. cerevisiae msh2∆* mutants have been exploited to improve our understanding of meiotic recombination mechanisms in an engineered polymorphic region harbouring a hotspot (Ahuja et al. 2021) and on a genome-wide scale (Martini et al. 2011; Marsolier-Kergoat et al. 2018; Cooper et al. 2021). This approach uncovered molecular details of non-Mendelian events during meiotic recombination at an unprecedented resolution (see below), but this also has its limitations because deletion of *mutS*α (and *mutL*α) genes does also affect overall gene conversion and crossover frequency (Martini et al. 2011; Brown et al. 2019; Ahuja et al. 2021; Cooper et al. 2021).

## How do we interpret the genetic and molecular evidence?

hDNA tracts which subsequently manifest as PMS and gene conversion events can be associated with crossovers and non-crossovers (see above). All these events are initiated by programmed DSBs, but at which point in their further processing do the repair pathways leading to either crossovers or non-crossovers diverge? Answering this question will enable a detailed understanding of how given recombination outcomes are generated, which has profound implications for interpreting genetic data. Early models of meiotic recombination argued that crossovers and non-crossovers are produced by very different DSB repair pathways. Non-crossovers were considered to be the results of synthesis-dependent strand annealing (Fig. 2) (Resnick 1976), and both crossovers and non-crossovers were thought to be produced by the “DSB repair” pathway involving the formation of double Holliday junctions (Szostak et al. 1983). The name of the latter repair pathway is not ideal but has historical reasons. In these early models the resolution of Holliday junctions was proposed to be unbiased because these recombination intermediates are symmetric and would thus be processed into crossovers and non-crossovers at an equal rate. Over the years, these models were refined, as there were strands of evidence supporting an early divergence of non-crossover and crossover formation. Firstly, in various species the gene conversion tracts of crossovers are longer than that of non-crossovers (Mancera et al. 2008; Wijnker et al. 2013; Cole et al. 2014). Secondly, in *S. cerevisiae* non-crossover recombination intermediates arise earlier during meiosis than crossover ones (Allers and Lichten 2001). This led to the idea that non-crossovers are predominantly the result of synthesis-dependent strand annealing, whereas biased resolution of double Holliday junctions exclusively produces crossovers.

**Fig. 4:**
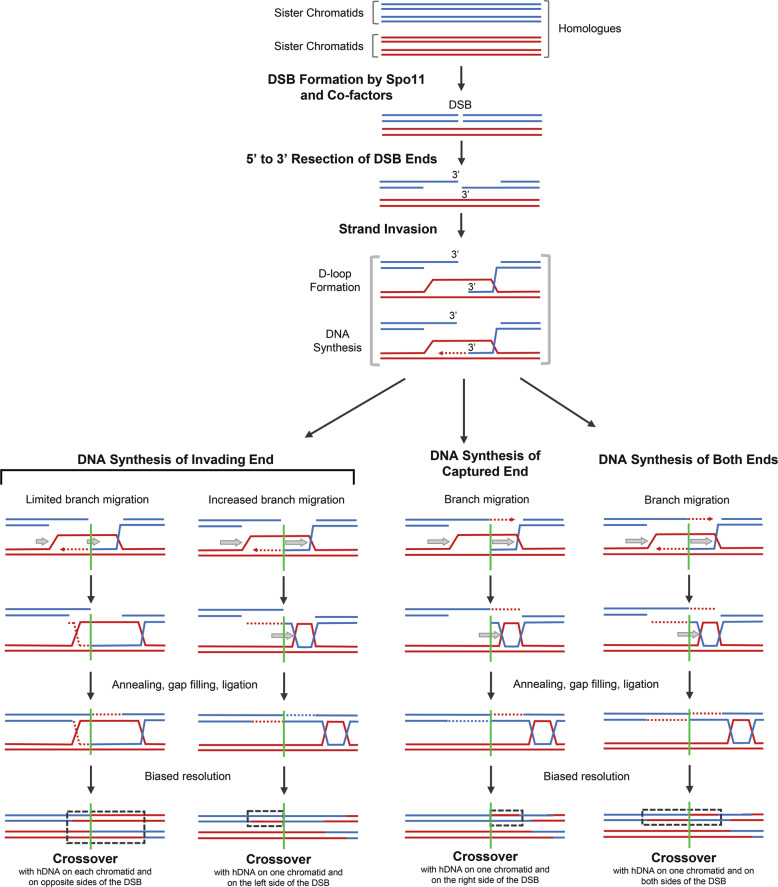
Models of crossover DSB repair pathways during meiosis. A homologous chromosome pair is represented by blue and red sister chromatids. For clarity only the chromatids involved in recombination are shown after the initial step of the pathways. Please, note that the small gap representing the DSB does not indicate loss of genetic material as with Spo11 double cutting (see Fig. 3). The early steps of recombination are shown as before (see Fig. 2). These include: DSB formation by Spo11, 5’ to 3’ resection of DSB ends on the broken DNA duplex (blue) to expose 3’ single-stranded tails, and strand invasion of the intact DNA duplex (red) to form a Displacement loop (D-loop). For crossover repair pathways, D-loop formation is then followed by DNA synthesis, helicase-mediated branch migration (grey arrows), capture of the second end of the DSB, further processing to produce double Holliday junctions, and biased resolution of these double Holliday junctions into crossovers. The position of the initiating DSB site is indicated by a green vertical line. **Left:** DNA synthesis of the invading end only, followed by limited branch migration and further processing, results in a crossover with hDNA in each chromatid and on opposite sides of the DSB. If branch migration is more extensive, moving the single-end invasion intermediate away from the initiating DSB site, resolution results in a crossover with hDNA on one chromatid and on the left side of the DSB. **Centre:** DNA synthesis of the captured end only, followed by branch migration and further processing, results in a crossover with hDNA on one chromatid and on the right side of the DSB. **Right:** DNA synthesis of both the invading end and the captured end, followed by branch migration and further processing, results in a crossover with hDNA on one chromatid and on both sides of the DSB. Note that branch migration is depicted as unidirectional (to the right) for simplicity but can occur in the opposite direction as well. Branch migration is also possible after annealing.

More recent experiments performed in *S. cerevisiae* revealed that non-Mendelian segregation tracts associated with crossovers and non-crossovers are considerably more complex than originally appreciated, but also more similar to one another (Figs. 2 and 4) (Martini et al. 2011; Oke et al. 2014; Marsolier-Kergoat et al. 2018; Ahuja et al. 2021). Also, in *Drosophila melanogaster*, conversion tracts in crossovers and non-crossovers are of similar length (Comeron et al. 2012), and display discontinuities in a *Msh6* mutant (Radford et al. 2007). More importantly, many of the observed complexities in the hDNA are incompatible with non-crossovers and crossovers being generated by the simple models of synthesis-dependent strand annealing and DSB repair involving Holliday junctions, respectively. A unified hypothesis, the Disassembly/Migration-Annealing (D/M-A) model, addresses these issues (Lao et al. 2008; Ahuja et al. 2021). This D/M-A model basically suggests that both crossovers and non-crossovers are formed by synthesis-dependent strand annealing, which generates non-Mendelian segregation tracts (hDNA) in the process, and only subsequent events stabilise certain recombination intermediates to create double Holliday junctions (Ahuja et al. 2021). The resolution of these double Holliday junctions then predominantly, if not exclusively, results in crossovers (Fig. 4).

## Which genetic factors are involved?

Are non-Mendelian segregation events (gene conversions) genetically separable from Mendelian ones (crossovers)? The short answer is, not really, apart from one partial exception. Genetic factors which direct or influence the formation and repair of DSBs will affect the frequency of both non-Mendelian and Mendelian segregation events, because these genetic outcomes are determined by the amount and type of meiotic recombination occurring at any given locus. Even the mismatch repair factors MutSα (Msh2-Msh6), Mutsβ (Msh2-Msh3), and MutLα (Mlh1-Pms1), which deal with mismatches in hDNA to produce gene conversions, and modulate the number of non-Mendelian segregation events at polymorphic sites by antirecombination, do also influence overall crossover frequency (Martini et al. 2011; Brown et al. 2019; Ahuja et al. 2021; Cooper et al. 2021). Having said this, the frequency of gene conversion and crossover outcomes can be differentially affected by experimental conditions and certain mutant backgrounds (Rockmill et al. 2013; Brown et al. 2020). This can be explained by changes in DSB frequency and in the way these DSBs are subsequently repaired into crossovers and non-crossovers (Rockmill et al. 2013; Protacio et al. 2022).

The only notable exception of a dedicated factor controlling non-Mendelian segregation is the MutLβ (Mlh1-Mlh2) complex in *S. cerevisiae* which restricts the tract lengths of gene conversion and PMS in cooperation with the meiosis-specific DNA helicase Mer3, in the context of both crossovers and non-crossovers (Duroc et al. 2017). The authors hypothesise that limiting the length of non-Mendelian segregation events might be necessary to avoid genome integrity issues, due to the high number of DSBs which are simultaneously made and repaired during meiosis (Duroc et al. 2017). They also suggest that too much gene conversion could be detrimental to the genetic diversity within a sexually reproducing population in the long run, as it might destroy favourable allele combinations and, especially in mammals, could cause the extinction of hotspot sequences (Cole et al. 2012; Duroc et al. 2017).

## Conclusion

There are many aspects about the generation of non-Mendelian and Mendelian meiotic events that are still enigmatic. The extent to which the mechanism(s) governing non-Mendelian segregation events, gene conversion and PMS, are evolutionarily conserved is also not fully elucidated. There are clear differences between species. For example, gene conversion tracts in *S. cerevisiae* are substantially longer than in multicellular eukaryotes, and some factors regulating features of non-Mendelian events in *S. cerevisiae* are not conserved in *Sz. pombe* (Mlh2, Mer3). The large evolutionary distance between the two model yeasts and their usefulness as experimental systems in meiosis research could be exploited comparatively to shed light on the conservation of the mechanisms enabling non-Mendelian segregation. Clearly, differences between homologous chromosomes in individuals within natural populations, influence where gene conversion events occur during meiosis, and whether they can be detected. This then also affects whether and how they contribute to genetic diversity in a given population. It will be important to bridge the knowledge gaps between molecular genetics, population genetics, and evolutionary biology, to arrive at a truly integrated model of meiotic recombination and its long-term role in generating genetic diversity.
